# Stroke in malignancy: complexities of diagnosis and management: a case report

**DOI:** 10.1186/s13256-019-2183-8

**Published:** 2019-08-20

**Authors:** Pakeeran Siriratnam, Thomas Kraemer, Ramesh Sahathevan

**Affiliations:** 1 Ballarat Health Services, 1 Drummond Street North, Ballarat, Victoria 3350 Australia; 2 Deakin University, Geelong, Victoria Australia; 3 University of Melbourne, Melbourne, Victoria Australia; 4 Florey Institute, Parkville, Victoria Australia

## Abstract

**Background:**

Although there is an established association between cancer and stroke, the role of malignancy as a causative agent or comorbidity is not always clear. Moreover, there are no established guidelines on the acute treatment of cancer-associated stroke or optimal anticoagulation. This case report illustrates the significance of these practice gaps.

**Case presentation:**

A 62-year-old Caucasian woman presented to our institute with acute neurological deficits and was found to have an occluded left middle cerebral artery on a computed tomographic angiogram. She was administered intravenous alteplase and underwent unsuccessful endovascular clot retrieval. Besides smoking and her age, she had no cerebrovascular risk factors, and the results of baseline investigations for the cause of stroke were negative. Subsequent computed tomography of the chest, abdomen, and pelvis showed metastatic malignancy, and in the context of a significantly elevated serum cancer antigen 19-9, we suspected a pancreatic primary cancer. A transthoracic echocardiogram demonstrated mitral regurgitation but no visible vegetation. The patient died of her illness. We made a diagnosis of cancer-associated stroke, specifically a likely case of nonbacterial thrombotic endocarditis.

**Conclusions:**

This case highlights the importance of having a high threshold of suspicion for malignancy as a cause of stroke.

## Background

Malignancy is a prothrombotic and hypercoagulable state, and it frequently presents as venous thrombosis. However, arterial thromboses are increasingly recognized in malignancy and may present as ischemic stroke. The prevalence of arterial embolic events is as high as 4.7% in patients with cancer [1]. Conversely, some studies suggest that around 10% of all patients with ischemic stroke have comorbid cancer. These figures are likely to increase in the future with a significant rise in cancer incidence and survival [2]. Similar to venous thromboembolism, arterial thromboembolism is more common in pancreatic, gastric, and lung adenocarcinomas [3]. Interestingly, the risk of strokes is higher in the early stages of these malignancies, reflecting a markedly increased immediate risk of arterial thromboembolism [4].

The treatment of stroke is rapidly evolving. The term *hyperacute management of stroke* encompasses thrombolysis and mechanical thrombectomy, frequently in combination [5]. However, even in this acute setting, a knowledge of the patient’s medical history plays a vital role. The prompt recognition of the association of stroke and malignancy is crucial in the acute and long-term management of stroke, but it is not always clear. This case report illustrates the complexities surrounding the diagnosis and management of a patient with cancer-associated stroke.

## Case presentation

A 62-year-old Caucasian woman presented to our institute with acute right hemiparesis, neglect, and aphasia (National Institutes of Health Stroke Scale score of 24). On examination, her blood pressure was 129/74 mmHg, pulse 84 beats per minute and regular, and temperature 36.8 °C. She had global right-sided weakness with a power grading of 1/5, with increased tone and brisk reflexes on the upper and lower limbs. Her plantar reflexes were downgoing. The examination of the left side of her body was unremarkable. It was not possible to assess for sensory, proprioceptive, and vibration changes, given her expressive and likely receptive dysphasia. She also had a pansystolic murmur audible in the mitral area, and her lungs were clear to auscultation. Her calves were soft and nontender bilaterally, and no peripheral edema was present. She was treated with intravenous alteplase and transferred to a tertiary center for endovascular clot retrieval (ECR) within 2 hours of her initial presentation. She was transferred back to our institute for ongoing care 3 days later. Once back under our care, we were able to confirm that she had no significant cardiovascular risk factors except for cigarette smoking of unknown pack-years and her age. She did not consume alcohol regularly. She had no similar or significant family, social, environmental, or employment history. She was receiving no regular medications prior to her stroke. We were also made aware that she was being investigated for a possible malignancy of undetermined source. The history was based on review of her medical records and collateral information provided by her family.

**Fig. 1:**
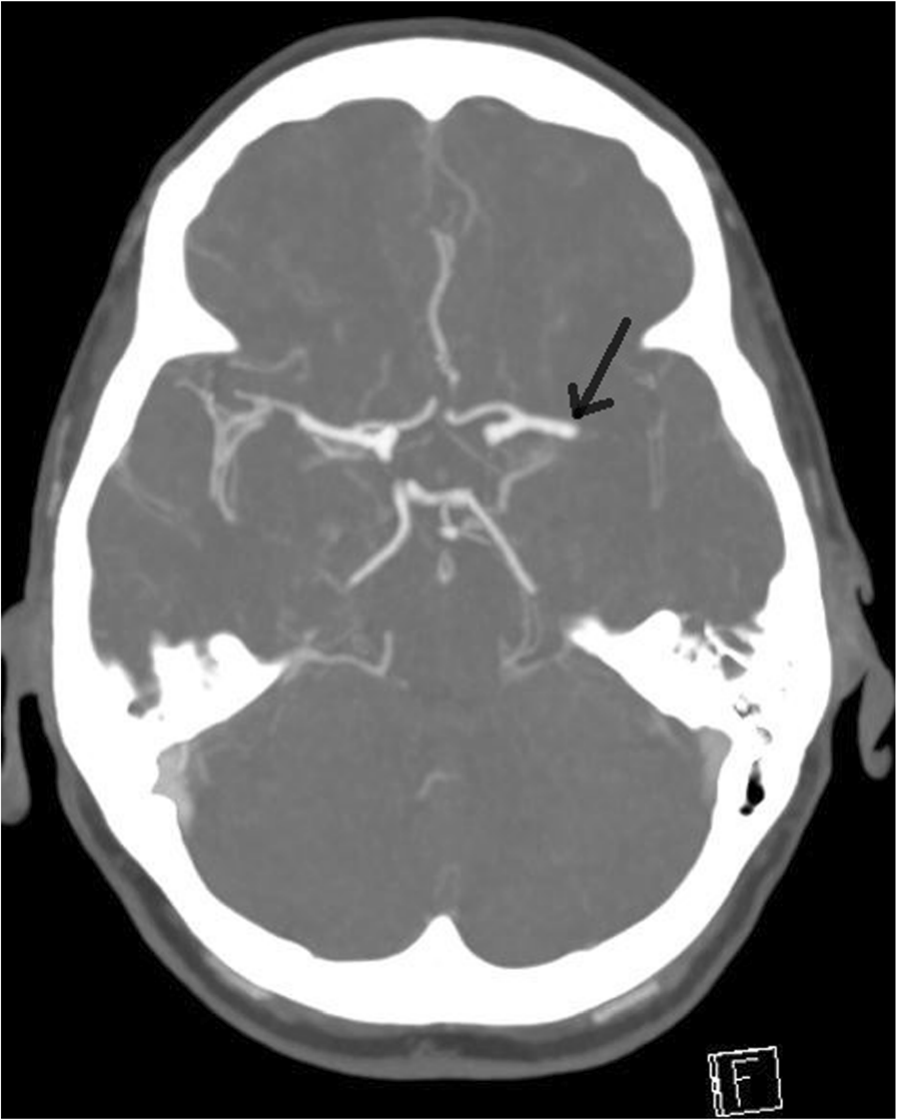
CTA done during the stroke call, demonstrating filling defect in the left proximal MCA (arrowed)

An immediate computed tomographic angiogram of her brain showed an occlusion of the proximal M1 segment of the left middle cerebral artery (MCA) (Fig. 1). Her cerebral vasculature was otherwise normal. Investigations assessing for vascular risk factors and secondary causes of stroke returned normal results. This included screening for underlying autoimmune disease and coagulopathy. The results of liver and renal function tests, electrolytes, and full blood counts were all within normal limits. Her fasting lipids and blood sugars were also normal. She had normal telemetry (≥ 72 hours) and imaging of the aortic arch and carotid arteries. She had no evidence of significant atherosclerosis or stenosis. A transthoracic echocardiogram (TTE) revealed mild to moderate mitral regurgitation with no vegetation or other sources of emboli.

**Fig. 2:**
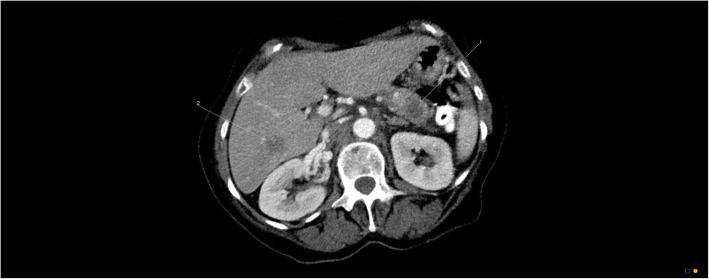
CT chest/abdomen/pelvis showed widespread metastases. Left arrow points to hepatic lesion, and right arrow demonstrates the pancreatic mass

**Fig. 3:**
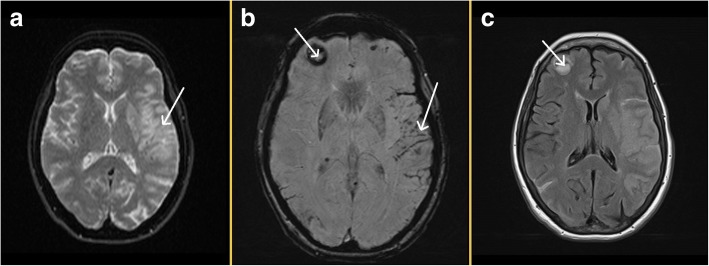
**a**: Established infarct in the left MCA seen in the DWI sequence, indicated by the arrow **b**: Right sided arrow demonstrates luxary hyperperfusion in the area of the infarct without significant hemorrhagic transformation on the SWI sequence, and left sided arrow shows a cerebral mass **c**: Flare sequence favours a probable cerebral metastatic deposit (arrowed)

Further imaging revealed metastases to the lung and liver with a pancreatic mass and retroperitoneal lymphadenopathy (Fig. 2). Her serum cancer antigen 19-9 (CA 19-9) was 1650 U/ml (reference range < 38 U/ml). Follow-up imaging of the brain showed an established infarct of the left MCA (Fig. 3a) with evidence of luxury perfusion within the infarct but no frank hemorrhagic transformation (Fig. 3b). A contrast-enhanced study of the brain showed evidence of probable cerebral metastases but not within the area of infarct (Fig. 3c).

Our patient had a stroke of undetermined mechanism. The imaging studies suggested a thromboembolic pattern, but no source of clot was identified in the heart or major arteries. She likely had an undiagnosed metastatic adenocarcinoma of the pancreas, based on her imaging and CA 19-9 levels, although we did not have the benefit of a tissue diagnosis. Screening for breast, ovarian, and colorectal carcinomas was negative. We suggest that a possible mechanism of stroke in our patient was an embolus arising centrally. The patient’s TTE showed evidence of mitral valve disease but no obvious vegetation. A transesophageal echocardiogram (TOE) would have been preferred, but the patient was not clinically stable enough to undergo the examination.

Treatment with thrombolysis and ECR was unsuccessful in our patient. We commenced her on secondary stroke prevention, as well as low-molecular-weight heparin (LMWH) for prevention of further thromboembolic events. In addition to the 67.5 mg of alteplase that she received, she was also started on aspirin 100 mg daily, amlodipine 5 mg daily, perindopril 5 mg daily, atorvastatin 80 mg daily, fluoxetine 20 mg daily, and enoxaparin 70 mg twice daily. She had ongoing physiotherapy, occupational therapy, and speech evaluation. Once she was diagnosed with metastatic malignancy, we consulted the oncology team and held a family meeting to discuss treatment options. Based on the family’s request and their opinion about the patient’s wishes, a tissue diagnosis was sought, albeit unsuccessfully.

The patient’s condition continued to decline, and she refused further treatment. Comfort measures were initiated following discussion with her family, and she died approximately 1 month following the stroke. An autopsy was not performed, in accordance with the family’s wishes.

## Discussion and conclusions

Our patient was a middle-aged woman with a stroke of undetermined mechanism found to have a metastatic malignancy of unknown primary. Our case highlights the importance and implications of diagnosing strokes driven by malignancy, as well as the practical difficulties clinicians face in managing an increasingly common condition that still has no clear guidelines.

The prothrombotic nature of cancer, attributed to the ability of tumor cells to produce antithrombotic and procoagulant substances and inflammatory cytokines, is considered a significant cause of stroke in malignancy. Tumors release microparticles into the blood, increase procoagulant factors, or release mucin, each of which activates platelets and endothelial cells. Moreover, cancer stimulates neutrophils to release decondensed chromatin, resulting in neutrophil extracellular traps, which promote further thrombosis [6].

The major mechanisms through which cancer can lead to stroke are hypercoagulability, nonbacterial thrombotic endocarditis (NBTE), direct compression of cranial blood vessels by the tumor, or cancer treatment [6]. Another plausible mechanism for stroke in the setting of cancer is paradoxical embolism in the presence of a patent foramen ovale and a right-to-left shunt. Other, rarer mechanisms include atrial fibrillation, septic embolism, tumor embolism, intravascular coagulation, hyperviscosity, and cerebral vein thrombosis. The exact pathophysiology of cancer in stroke mostly remains undetermined and is more likely a combination of these factors [3].

Large artery and small vessel disease are estimated to account for 25–33% of strokes in patients with a malignancy. In addition to the shared risk factors, cancer independently promotes atherosclerotic plaque formation and systemic inflammation. Cryptogenic strokes account for around 30% of stroke in the general population but are responsible for approximately 50% of cancer-associated strokes [6]. However, some studies suggest that cancer is responsible for 20% of cryptogenic strokes [7]. Autopsy studies suggest that NBTE is the leading cause of cryptogenic strokes in malignancy [5].

NBTE, sometimes referred to as *marantic endocarditis*, was first described by Zeigler in 1888, but the term *nonbacterial thrombotic endocarditis* was only coined by Gross and Friedberg in 1936 [8, 9]. NBTE is marked by sterile vegetations on the cardiac valves, thought to be secondary to disrupted fibrin attached to cardiac valves in high-flow areas, which then act as a network for platelets to adhere to. The mitral and aortic valves are the most common sites of vegetation, being exposed to higher hemodynamic pressure [10]. These vegetations may dislodge and cause occlusion within the arterial tree, leading to stroke, among other sequelae [8]. NBTE is most commonly associated with adenocarcinomas [9]. The mucin produced by adenocarcinomas induce hypercoagulability by activating L- and P-selectins, which stimulate leukocytes to trigger formation of platelet-rich microthrombi. These microthrombi subsequently activate the CD150 receptors in leukocytes, monocytes, and endothelium, leading to an increased production of tissue factor and subsequent thrombus formation [11]. The incidence of thromboembolic disease in pancreatic cancer ranges from 17% to 57% [12].

There are no pathognomonic clinical features of stroke due to NBTE, but cortical stroke is more likely, which can lead to deficits of higher function, such as neglect and aphasia [13]. The presence of a heart murmur or valvular abnormality on imaging, other manifestations of systemic embolization, and the presence of an associated disease process such as malignancy are indicators of the diagnosis [8]. Echocardiogram is an important tool in the diagnosis of NBTE. TTE is frequently nondiagnostic, and although TOE has greater sensitivity, it is less frequently used [9, 14, 15]. The spectrum of gross pathology in NBTE ranges from visible vegetations on the cardiac valves, to valvular dysfunction in the absence of visible vegetation, to degenerative changes only appreciated on histopathology. Therefore, absence of vegetations even on a TOE does not exclude NBTE [14]. Furthermore, in pancreatic cancer, the vegetations are often too friable and small to be identified on an echocardiogram [10]. The mitral regurgitation on our patient’s echocardiogram may have been the result of gross pathology not appreciated on the TTE.

Cryptogenic strokes in patients with cancer, compared with patients without cancer, are more likely to be associated with elevated D-dimer levels and involve multiple vascular territories [7, 16]. Suggested indications to screen for occult malignancies in patients with cryptogenic stroke are when traditional risk factors do not adequately explain the stroke, atypical presentation, and multiterritorial involvement [7].

Current guidelines on hyperacute stroke care do not directly reference stroke in malignancy [2]. There is mixed evidence on the efficacy and safety of thrombolysis in the setting of stroke [3, 17–19]. In spite of this, the use of thrombolysis in patients with cancer and stroke has increased significantly in recent times, although rates of use are one-third less than in patients with non-cancer-related ischemic strokes [3]. The 2018 American Stroke Association guidelines do not list active cancer as an absolute contraindication for thrombolysis, despite its efficacy and safety not being well established [20]. Although there are no trial data on the utility of ECR in patients with cancer, some studies have found similar rates of intracerebral hemorrhage after mechanical thrombectomy in patients with and without cancer [3].

Preventing recurrent thromboembolism is a critical therapeutic goal in cancer-associated strokes and involves treating the underlying pathology and antithrombotic therapy [10]. However, there are again no clear guidelines on the optimal anticoagulant [2, 3]. Although there is no consensus on the preference between LMWH and unfractionated heparin, both are recommended over warfarin in the treatment of malignancy-induced hypercoagulable states [8, 10, 21]. Recent evidence indicates a potential role for direct oral anticoagulants (DOACs) in patients with cancer with venous thrombosis, but more evidence is needed before widespread use of DOACs in malignancy becomes standard practice [3, 22]. The current secondary prevention for venous thromboembolism in malignancy recommends LMWH for a minimum of 6 months [23]. It is important to note, however, that this relates to venous events, and there is a paucity of data relating to arteriothromboembolic events. The role of antiplatelets as secondary prevention in this setting is also unknown [6]. A recent randomized controlled trial compared the use of enoxaparin with aspirin in patients with cancer-related ischemic stroke and found no difference in terms of major bleeding, thromboembolic events, or survival [24]. This study was limited by low participant numbers, and requires larger trials to verify their results. As identified by the American Stroke Association and the European Stroke Organisation, the optimal antithrombotic regimen in cancer-associated strokes requires further high-powered, randomized studies [20, 25].

This case report serves as a reminder that health care professionals should consider malignancy as a potential cause of cryptogenic stroke. There is an established association between NBTE and adenocarcinomas, and an absence of vegetations on an echocardiogram does not exclude the condition. A limitation of this case report is the inability to confidently determine the mechanism, given that we did not have the benefit of tissue diagnosis to prove the primary malignancy and did not perform lower limb ultrasound and bubble study to exclude paradoxical embolism. However, our early recognition of the underlying pathology provided us with vital prognostic information in guiding the extent of investigations and management, which reflects a patient-centered approach and real-life practice. This case highlights the need for further research and clinical trials to guide the management of cancer-associated strokes. On the basis of current evidence, acute treatment with ECR and thrombolysis should not be disqualified. Parenteral heparin should be used in NBTE to prevent recurrent thrombosis.

## Data Availability

Not applicable.
